# Serum D-dimer levels at admission for prediction of outcomes in acute pancreatitis

**DOI:** 10.1186/s12876-019-0989-x

**Published:** 2019-05-02

**Authors:** Jianhua Wan, Xiaoyu Yang, Wenhua He, Yin Zhu, Yong Zhu, Hao Zeng, Pi Liu, Liang Xia, Nonghua Lu

**Affiliations:** Department of Gastroenterology, The First Affiliated Hospital of Nanchang University, 17 Yongwaizheng Street, Nanchang, Jiangxi 330006 People’s Republic of China

**Keywords:** Acute pancreatitis, D-dimer, Acute necrotic collection, Multiple organ failure, Prognosis

## Abstract

**Background:**

Systemic alterations in coagulation are associated with complications of acute pancreatitis (AP). D-dimer, a fibrin degradation product, was recently described as a marker of pancreatitis outcome. Early prediction is essential for reducing mortality in AP. The present study aims to assess the relationship between elevated serum D-dimer levels and the severity of AP.

**Methods:**

We performed an observational retrospective study with data from 3451 enrolled patients with AP. Serum D-dimer levels were measured upon admission, after 24 h and during the week after admission by immunoturbidimetry. Univariate and multivariate analyses were used to determine whether elevated D-dimer levels were independently associated with the severity of AP.

**Results:**

Of the 3451 AP patients, 2478 (71.8%) had serum D-dimer levels measured within 24 h of hospital admission; 1273 of these patients had D-dimer levels ≤2.5 mg/L, and 1205 had D-dimer levels > 2.5 mg/L (934 patients had mild AP (MAP); 1086, moderately severe AP (MSAP); and 458, severe AP (SAP)). Patients with D-dimer levels > 2.5 mg/L (*n* = 1205) had higher incidences of SAP (75.5% vs. 24.5%), acute peripancreatic fluid collection (APFC) (53.3% vs. 46.7%), acute necrotic collection (ANC) (72.4% vs. 27.6%), pancreatic necrosis (PN) (65.2% vs. 34.8%), infected pancreatic necrosis (IPN) (77.7% vs. 22.8%), organ failure (OF) (68.5% vs. 31.5%), persistent organ failure (POF) (75.5% vs. 24.5%), ICU requirement (70.2% vs. 29.8%), and mortality (79.2% vs. 20.8%) than did patients with D-dimer levels ≤2.5 mg/L (*n* = 1273). The multivariate analysis showed that patients with higher serum D-dimer levels had poorer prognoses that worsened over time.

**Conclusion:**

The measurement of D-dimer levels at admission may be useful for risk stratification of AP.

## Background

Acute pancreatitis (AP) is a common clinical acute abdominal disease that has a narrow therapeutic window. Currently, AP is classified as mild AP (MAP), moderately severe AP (MSAP), and severe AP (SAP) [1]. The majority of patients have a mild form of the disease and recover well, but approximately 20% develop SAP, which has a high mortality rate (15–35%) [2], mainly due to pancreatic necrosis, systemic inflammation and persistent multiple organ dysfunction associated with concurrent infections [3–5]. In addition, the annual incidence of AP has increased along with medical costs, and AP is one of the leading causes of in-hospital deaths in developed countries [3, 6–9]. Early treatment, for which the early diagnosis and assessment of AP severity are essential, has been shown to reduce mortality [10, 11]. Currently, however, no method of effectively detecting disease severity is available [12]. Various laboratory markers [13] have been used to predict AP severity; however, the low accuracy, cumbersome laboratory techniques and high cost associated with these approaches currently limit their clinical application. Furthermore, the existing scoring systems seem to have reached their maximal efficacy [14]. Performing CT upon admission solely to assess AP severity is not recommended [15], and radiological tests are expensive to perform. Therefore, new biomarkers are desirable and necessary for better predicting the severity of AP.

Pancreatitis induces the formation of venous thrombosis [16–18]. Thrombosis is a vascular complication of AP and a major cause of AP morbidity and mortality [19]. Notably, in a recent study, Min-Jung Park observed intrapancreatic thrombosis [20]. The early peak in AP mortality is mainly due to the systemic inflammatory response; the circulatory system is in a hypercoagulable state, which may induce the formation of thrombosis and aggravate AP due to tissue ischemia. Abdulla A found that the depletion of platelets decreased cerulein-induced myeloperoxidase (MPO) levels and neutrophil recruitment in the pancreas [21]. Moreover, the administration of heparin alleviated cerulein-mediated pancreatic injury [20]. These studies suggested that thrombosis has a critical impact on the prognosis of AP. D-dimer, a soluble fibrin degradation product, is central to the diagnostic workup of suspected deep-vein thrombosis and pulmonary embolism [22]. Numerous studies have shown that D-dimer serves as a valuable marker of the activation of coagulation and fibrinolysis [23]. Furthermore, D-dimer levels are elevated in a variety of conditions, including atrial fibrillation [24], coronary artery disease [25], and HIV infection [26], suggesting that D-dimer may play an important role in the assessment of AP severity. The use of D-dimer levels as an indicator of disease severity in small samples of patients has recently been reported [27, 28], but the quality of the evidence is low. Thus, the association between D-dimer levels and AP severity requires further investigation.

The purposes of this investigation were to define the sensitivity of D-dimer levels for predicting AP severity with our data and to assess the potential of serum D-dimer levels as a marker of disease severity in AP patients.

## Methods

In this observational study, all consecutive patients were retrospectively collected from our single-center hospital between January 1, 2014, and December 31, 2017. The ethics committee of The First Affiliated Hospital of Nanchang University reviewed and approved this study (No. 2011001).

### Patient selection

We collected the data from an electronic medical database in our hospital. Patients who were diagnosed with AP and had serum D-dimer levels measured within 24 h of presentation were included in the study. The criteria for a diagnosis of AP include classic abdominal pain, serum amylase and/or lipase and radiographic evidence, which were described in a previous study [10]. The classification of acute pancreatitis is well recognized according to the latest 2012 revision of the Atlanta classification (MAP, SAP and MSAP).

### Data collection

For each patient, age, sex, medical history, admission number, and date were collected as baseline demographic data. Moreover, we collected vital signs of all patients on admission and important laboratory tests, radiological data and clinical outcomes after hospitalization.

### Statistical methods

IBM Statistical Package for Social Sciences (SPSS) software version 20.0 (Chicago, USA) was used to perform the statistical analyses. The results are presented as percentages (%) or means ± SD. Comparisons were performed using Student’s t test for two groups of independent samples, Cuzick’s trend test for multiple groups and the χ2 and Fisher’s exact tests for categorical variables. Logistic regression analyses were performed to predict risk factors with categorical dependent variables. Differences were considered to be statistically significant at *P* < 0.05.

## Results

Of the 3451 patients with AP, 2478 (71.8%) had serum D-dimer levels measured within 24 h of hospital admission. The demographics and clinical characteristics (sex; age; body mass index (BMI); and common etiologies, including biliary dysfunction, hypertriglyceridemia and alcoholism) were similar between the patients with available early serum D-dimer levels (*n* = 2478) and those without (*n* = 973), as shown in Table 1. No significant differences were observed between the patients with available early serum D-dimer levels and those without in terms of clinical outcomes, including APFC (26.4% vs. 28.0%), ANC (17.4% vs. 15.4%), PN (22.3% vs. 24.4%), POF (18.5% vs. 17.0%), and mortality (2.0% vs. 1.2%; all *P* > 0.1; Table 1).

**Table 1:** Comparison of baseline clinical characteristics and outcomes between AP patients with vs. without serum D-dimer drawn in admission to the hospital

	D-dimer measured	D-dimer not available	
Variables	*N* = 2478	*N* = 973	*P*
Male, *N* (%)	1405 (56.7)	510 (52.4)	0.492
Median age, (IQR)	51 (40–64)	51 (40–64)	0.759
Median BMI, (IQR)	23 (21–25)	23 (21–25)	0.838
Etiology, *N* (%)			0.309
Biliary	1448 (58.4)	587 (60.3)	
Alcoholism	214 (8.6)	70 (7.2)	
Hypertriglyceridemia	646 (26.1)	265 (27.2)	
Others	170 (6.9)	51 (5.2)	
Outcomes			
APFC, *N* (%)	653 (26.4)	272 (28.0)	0.314
ANC, *N* (%)	431 (17.4)	150 (15.4)	0.163
PN, *N* (%)	552 (22.3)	237 (24.4)	0.190
POF, *N* (%)	458 (18.5)	165 (17.0)	0.197
Mortality, *N* (%)	50 (2.0)	12 (1.2)	0.119

*AP* acute pancreatitis, *N* number, *APFC* acute peripancreatic fluid collection, *ANC* acute necrotic collection, *PN* Pancreatic necrosis, *POF* persistent organ failure, *IQR* Inter Quartile Range

**Fig. 1:**
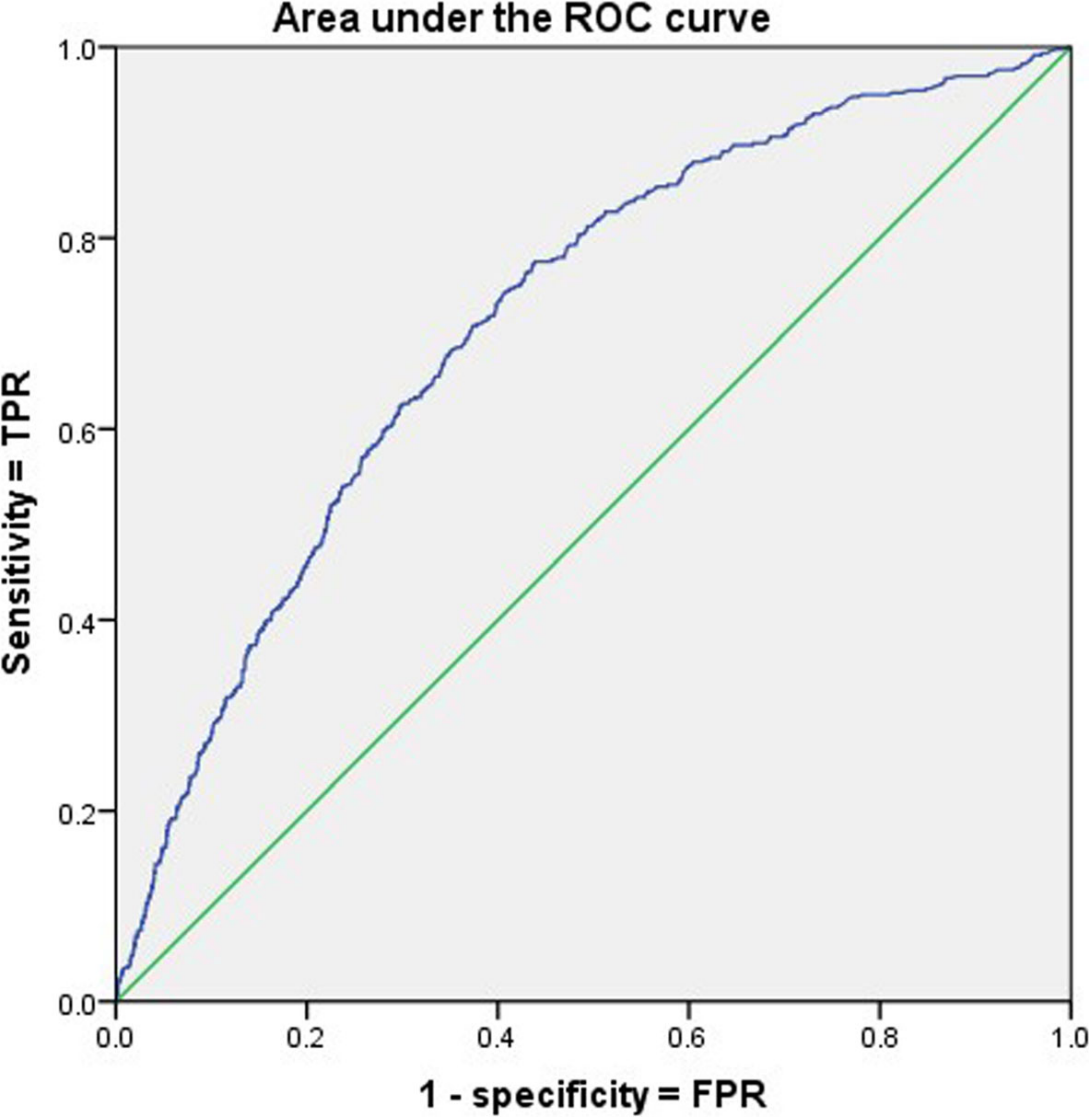
The area under the curve (AUC) of serum D-dimer levels to predict SAP

For the prediction of SAP, the area under the curve (AUC) for serum D-dimer levels was 0.714 (*P* < 0.001; Fig. 1). To calculate the accuracy of the serum D-dimer levels, an optimal cutoff value of 2.5 mg/L was used for the prediction of SAP, as shown in Table 2. The 1205 patients with a serum D-dimer level above 2.5 mg/L demonstrated a poorer prognosis than the 1237 patients with a serum D-dimer level below 2.5 mg/L (Table 2). Higher incidences of SAP (75.5%), APFC (53.3%), ANC (72.4%), PN (65.2%), IPN (77.7%), OF (68.5%), POF (75.5%), ICU requirement (70.2%), and mortality (79.2%) were observed in the high D-dimer group, and longer median hospital stays (7 vs. 10) and median ICU stays (0 vs. 3) were found in the high D-dimer group.

**Table 2:** Population baseline characteristics between serum D-dimer level categories

	D-dimer ≤2.5	D-dimer > 2.5	
Variable	N = 1273	N = 1205	*P*
Severity classification, *N* (%)			< 0.001
MAP	667 (52.4)	267 (22.2)	
MSAP	494 (38.8)	592 (49.1)	
SAP	112 (8.8)	346 (28.7)	
APFC, *N* (%)	305 (24.0)	348 (28.9)	0.005
ANC, *N* (%)	119 (9.3)	312 (25.9)	< 0.001
PN, *N* (%)	192 (15.1)	360 (29.9)	< 0.001
Infected PN, *N* (%)	31 (2.4)	108 (9.0)	< 0.001
OF, *N* (%)	258 (20.3)	561 (46.6)	< 0.001
POF, *N* (%)	112 (8.8)	346 (28.7)	< 0.001
Median hospital days, (IQR)	7 (5–10)	10 (6–16)	< 0.001
Median ICU days, (IQR)	0 (0–2)	3 (0–7)	< 0.001
Admission to ICU, *N* (%)	203 (15.9)	479 (39.8)	< 0.001
Mortality, *N* (%)	10 (0.8)	38 (3.2)	< 0.001

*N* number, *MAP* mild acute pancreatitis, *MSAP* moderately severe acute pancreatitis, *SAP* severe acute pancreatitis, *APFC* acute peripancreatic fluid collection, *ANC* acute necrotic collection, *PN* Pancreatic necrosis, *OF* organ failure, *POF* persistent organ failure, *IQR* Inter Quartile Range

The serum D-dimer level was recorded on different days within 1 week of admission to observe sequential changes in this indicator during the early days following admission to the hospital with AP. The overall D-dimer level gradually increased after admission. In the analysis of AP severity classification, the D-dimer level in the SAP group gradually increased but remained higher than that in the MSAP and MAP groups (all P-trend< 0.001; Table 3). Similarly, the outcomes of AP, including organ failure and pancreatic necrosis, worsened with higher levels of serum D-dimer (all *P* < 0.01; Table 3). Thus, the serum D-dimer level seems to be a useful marker for severity classification and outcomes in AP.

**Table 3:** Table showing D-dimer levels in various groups on different days of admission to the hospital within 1 week

	Day 1	Day 2	Day 3	Day 5	Day 7
Variable	N = 2478	*N* = 1413	*N* = 604	*N* = 558	*N* = 385
Severity classification					
MAP	2.69 ± 5.58	2.70 ± 4.41	3.69 ± 4.65	4.03 ± 6.34	5.66 ± 6.42
MSAP	4.46 ± 5.29	5.09 ± 6.23	5.59 ± 4.96	6.69 ± 5.48	7.00 ± 5.41
SAP	7.21 ± 8.90	7.21 ± 7.09	8.03 ± 6.66	9.55 ± 8.93	11.10 ± 11.49
*P*_trend_	< 0.001	< 0.001	< 0.001	< 0.001	< 0.001
Organ failure					
NO	3.30 ± 5.20	3.53 ± 4.65	4.47 ± 4.64	5.61 ± 5.61	6.17 ± 4.77
YES	6.33 ± 7.98	6.76 ± 7.56	7.42 ± 6.32	8.70 ± 8.29	10.36 ± 10.64
*P*	< 0.001	< 0.001	< 0.001	< 0.001	< 0.001
Pancreatic necrosis					
NO	3.96 ± 6.56	4.36 ± 5.99	5.57 ± 6.08	6.52 ± 6.69	7.26 ± 7.08
YES	5.51 ± 6.11	5.99 ± 6.46	6.91 ± 5.04	9.26 ± 8.52	10.93 ± 11.07
*P*	< 0.001	< 0.001	0.007	< 0.001	< 0.001

*N* number, *MAP* mild acute pancreatitis, *MSAP* moderately severe acute pancreatitis, *SAP* severe acute pancreatitis

A comparison of the baseline clinical characteristics of AP patients by severity classification (MAP vs. MSAP vs. SAP) indicated that the proportion of men in each group was not significantly different (56.2% vs. 57.9% vs. 54.8%). SAP patients were older and weighed more than either MSAP or MAP patients. An etiology of hypertriglyceridemia was associated with a higher incidence of MSAP (30.2%) and SAP (29.9%) (P-trend< 0.001) than that of MAP. Glucose (GLU), blood urea nitrogen (BUN), and creatinine levels increased as AP severity increased (P-trend< 0.001; P-trend< 0.001; P-trend< 0.001). Furthermore, prothrombin time (PT) and activated partial thromboplastin time (APTT), as markers of blood coagulation, increased as AP severity increased (P-trend< 0.001; P-trend< 0.001). The number of cases (100; 21.8%) of infected pancreatic necrosis observed in the SAP group was higher than that observed in the MSAP group (3.5%) (P-trend< 0.001). Moreover, a higher incidence of mortality was observed in the SAP group than in the MSAP and MAP groups (9.8% vs. 0.3% vs. 0%; P-trend< 0.001), as shown in Table 4.

**Table 4:** Comparison of baseline clinical characteristics and outcomes between AP patients on severity classification

	MAP	MSAP	SAP	
Variable	*N* = 934	*N* = 1086	*N* = 458	*P*_trend_
Male, *N* (%)	525 (56.2)	629 (57.9)	251 (54.8)	0.492
Median age, (IQR)	51 (41–64)	49 (39–62)	55 (44–66)	< 0.001
BMI	22.74 ± 3.38	23.55 ± 3.54	23.77 ± 3.56	< 0.001
Etiology, *N* (%)				
Biliary	569 (60.9)	603 (55.5)	276 (60.3)	0.034
Alcoholism	67 (7.2)	104 (9.6)	43 (9.4)	0.130
Hypertriglyceridemia	181 (19.4)	328 (30.2)	137 (29.9)	< 0.001
Smoker, *N* (%)	175 (18.7)	230 (21.2)	104 (22.7)	0.178
Alcoholism, *N* (%)	155 (16.6)	224 (20.6)	112 (24.5)	0.002
GLU, mmol/L	7.17 ± 3.05	8.59 ± 4.13	9.81 ± 5.22	< 0.001
BUN, mmol/L	5.02 ± 2.88	5.61 ± 3.09	9.71 ± 7.37	< 0.001
Creatinine, mmol/L	67.37 ± 51.77	69.07 ± 42.42	130.85 ± 128.10	< 0.001
PT, s	12.21 ± 7.60	12.62 ± 6.69	13.97 ± 7.60	< 0.001
APTT, s	29.61 ± 10.58	31.41 ± 14.81	36.13 ± 14.40	< 0.001
D-dimer, mg/L	2.69 ± 5.88	4.46 ± 5.29	7.21 ± 8.90	< 0.001
Median APACHEII, (IQR)	5 (3–8)	6 (4–9)	10 (8–13)	< 0.001
PN, *N* (%)	0	328 (30.2)	220 (48.0)	< 0.001
Infected PN, *N* (%)	0	38 (3.5)	100 (21.8)	< 0.001
Median hospital days (IQR)	6 (4–8)	9 (6–14)	14 (9–25)	< 0.001
Median ICU days, (IQR)	0 (0)	0 (0–4)	7 (4–15)	< 0.001
Admission to ICU, N (%)	74 (7.9)	259 (23.8)	349 (76.2)	< 0.001
Mortality, *N* (%)	0 (0)	3 (0.3)	45 (9.8)	< 0.001

*MAP* mild acute pancreatitis, *MSAP* moderately severe acute pancreatitis, *SAP* severe acute pancreatitis, *N* number, *GLU* blood glucose, *BUN* Blood urea nitrogen, *PT* prothrombin time, *APTT* activated partial thromboplastin time, *PN* Pancreatic necrosis, *IQR* Inter Quartile Range

The logistic multivariate regression analysis showed an association between serum D-dimer level and complications after adjusting for age, sex, pancreatitis etiology, smoking status, and alcohol use status. As shown in Table 5, a higher serum D-dimer level was independently associated with pancreatitis prognosis and complications, including APFC, ANC, pancreatic necrosis, infected pancreatic necrosis, organ failure, persistent organ failure, ICU admission, and mortality.

**Table 5:** Multivariate analysis showing association of serum D-dimer level with complications after adjusting for age, sex, pancreatitis etiology, smoker, alcoholism

Complication	B	OR	*P*
APFC	0.27	1.30	0.004
ANC	1.25	3.49	< 0.001
PN	0.90	2.46	< 0.001
Infected PN	1.37	3.94	< 0.001
OF	1.21	3.35	< 0.001
POF	1.32	3.74	< 0.001
Admission to ICU	1.23	3.41	< 0.001
Mortality	1.34	4.04	< 0.001

*B* regression coefficient, *APFC* acute peripancreatic fluid collection, *ANC* acute necrotic collection, *PN* Pancreatic necrosis, *OF* organ failure, *POF* persistent organ failure

## Discussion

This study is a single-center observational retrospective analysis that evaluated simple laboratory parameters as predictors of SAP. Here, we studied the diagnostic value of D-dimer levels for predicting AP severity. Many indicators are currently available for predicting SAP and include C-reactive protein (CRP) and BUN, the most widely used parameters for the assessment of AP severity; however, none of them differ significantly within 24 h after the onset of symptoms. Levels of serum lipase and amylase, two major markers for pancreatitis, have also been shown to be disproportionate to the severity of the disease [8]. Thus, these indicators seem to have reached their maximal efficacy [2, 8, 13, 14, 29, 30]. New indicators for assessing the severity of AP have been reported in recent studies [28, 31–33]. However, these detection indicators are expensive and difficult to operate. In particular, the specific mechanism remains uncertain, and additional studies are needed. Interleukin-6 significantly improves the predictive value for severe acute pancreatitis but is difficult to detect [34]. With the advent of a fully automated assay, IL-6 is currently being used clinically in some hospitals [35]. Therefore, continuing to study valuable markers is necessary. The D-dimer level, which is a marker of the activation of coagulation and fibrinolysis, provides a rapid assessment of thrombotic activity and safely excludes patients with suspected venous thromboembolism (VTE) based on the clinical decision rule [36, 37]. In addition, D-dimer has been widely used in clinical settings because it is convenient and stable.

The possibility that D-dimer levels can predict the severity of AP may be explained by the following pathogenic mechanism. At the onset of AP, the abnormal activation of pancreatic enzymes results in inflammation and injury to the pancreas, which then induces thrombosis and further aggravates the injury [38]. Two mortality peaks occur in patients with AP, namely, early mortality due to the effects of systemic inflammatory response syndrome (SIRS) and multiple organ dysfunction syndrome (MODS) and late mortality caused by the effects of MODS combined with sepsis following pancreatic necrosis and infection. Systemic inflammation is a common risk factor for the development of VTE [22]. Clearly, crosstalk occurs between the inflammatory response and the clotting reaction. A disturbance of the coagulation system has long been thought to be implicated in the pathogenesis of systemic and local pancreatitis complications [18]. In addition to the thrombosis of the pancreas itself, pancreatitis leading to coronary venous thrombosis and splanchnic vein thrombosis have also been reported [39, 40]. D-dimer, a common indicator of thrombosis, may play an important role in the assessment of AP severity by monitoring pancreatic and extrapancreatic thrombosis. In our study, AP patients with high D-dimer levels had a higher incidence of pancreatic necrosis and organ failure than did patients with normal D-dimer levels. This result suggested that the severity of AP is closely associated with the presence of inflammation and thrombosis.

Few studies have been conducted to assess the value of D-dimer, a fibrin degradation product, in predicting AP severity. In those retrospective studies, patients with SAP had higher D-dimer levels than did those with MAP, suggesting that D-dimer levels had moderate diagnostic accuracy in predicting MSAP and exhibited excellent diagnostic accuracy in predicting SAP [27, 28, 41, 42]. In a single-center, retrospective study including 71 patients, Cécile Gomercic [41] obtained results implying that either alone or in combination with the CRP level, the D-dimer level may be a useful early predictive biomarker of AP. Aleksandra Boskovic [42] reported that the D-dimer level may be a simple clinical predictor of pediatric AP severity and local complications in a small cohort of 36 patients with AP.

Similar to these previous results, our results implied that the D-dimer level may be a potential biomarker for predicting AP severity. Compared with prior reports, our study has several strengths, including the continuous nature of patient enrollment and the large sample size. In addition, we recorded a sequential change in the serum D-dimer level in the early days (within 1 week) after admission to the hospital. The logistic multivariate regression analysis showed that the D-dimer level upon admission was independently associated with early prediction of AP outcome. In addition to the D-dimer level, the blood coagulation markers PT and APTT were found to increase with greater AP severity (P-trend< 0.001; P-trend< 0.001).

This study has several potential limitations. First, D-dimer could not be detected in some of the samples obtained upon admission; therefore, the measurement of D-dimer levels may be subject to selection bias. To counter this potential limitation, a head-to-head comparison of patients having available D-dimer values with those not having available D-dimer values was performed. No difference in clinical outcomes, including persistent organ failure, was found between the two groups. Second,

the D-dimer level is affected by many factors; any process (including pregnancy, inflammation, cancer, and surgery) that increases fibrin production or breakdown also increases D-dimer levels. In addition, D-dimer levels and age are closely related [43]. Furthermore, the magnitude of the unit of measure for the D-dimer level varies widely across laboratories using the same assays, and an accepted standardization for D-dimer concentration across assay types is lacking. The optimal D-dimer test methodology and associated threshold value for detecting AP remain uncertain. Consequently, the results available for one assay cannot simply be extrapolated to other assays, even those using similar formats [43, 44].

## Conclusions

In addition to excluding VTE, D-dimer testing may play a critical role in the prediction of AP severity. However, the wide variation in the types and operating characteristics of D-dimer assays means that the results of studies that used one assay cannot be extrapolated to studies that used another assay, which limits the use of multicenter studies. Much work remains to be done to standardize the performance and reporting of D-dimer assays as well as to translate the results of D-dimer studies into clinical practice.

## Abbreviations

ANC: Acute necrotic collection
AP: Acute pancreatitis
APFC: Acute peripancreatic fluid collection
APTT: Activated partial thromboplastin time
AUC: Area under the curve
BUN: Blood urea nitrogen
CRP: C-reactive protein
GLU: Glucose
IPN: Infected pancreatic necrosis
MAP: Mild AP
MODS: Multiple organ dysfunction syndrome
MPO: Myeloperoxidase
MSAP: Moderately severe AP
OF: Organ failure
PN: Pancreatic necrosis
POF: Persistent organ failure
PT: Prothrombin time
SAP: Severe AP
SIRS: Systemic inflammatory response syndrome
VTE: Venous thromboembolism
